# Supplementary material for: Interpretable GWAS by linking clinical phenotypes to quantifiable immune repertoire components
Source: Commun Biol. 2024 Oct 20;7:1357. doi: 10.1038/s42003-024-07010-x (PMC11491462; doi:10.1038/s42003-024-07010-x)
Supplement: Supplementary file 2 — Supplementary Information [file 42003_2024_7010_MOESM2_ESM.pdf]

Supplementary information

Interpretable GWAS by linking clinical phenotypes to quantifiable immune repertoire components

Yuhao Tan[1,2,3], Lida Wang[4], Hongyi Zhang[2,3], Mingyao Pan[2,3], Dajiang J. Liu[4,$], Xiaowei Zhan[5,$], Bo Li[1,2,3,$]

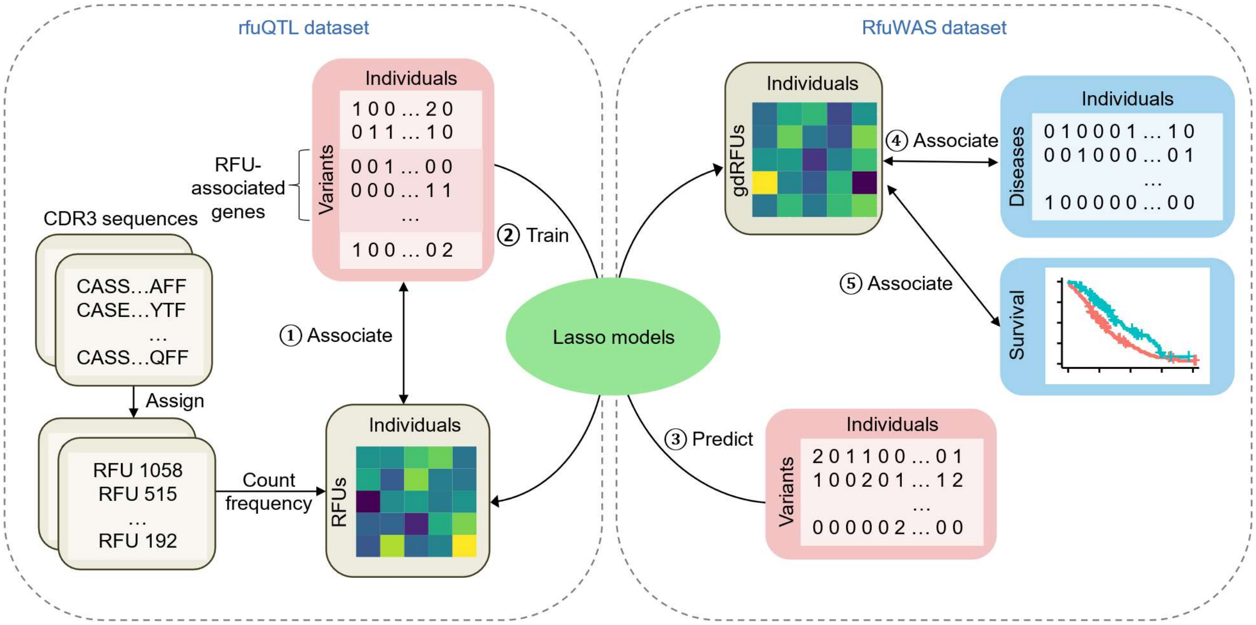

Supplementary Fig. 1 | Study design. We conducted step 1 and 2 in rfuQTL training set (n = 659), and conducted step 3, 4, and 5 in RfuWAS dataset (n = 337,122). Lasso models were trained to predict RFU abundances based on variants in RFU-associated genes. gdRFU, genetically determined RFU.

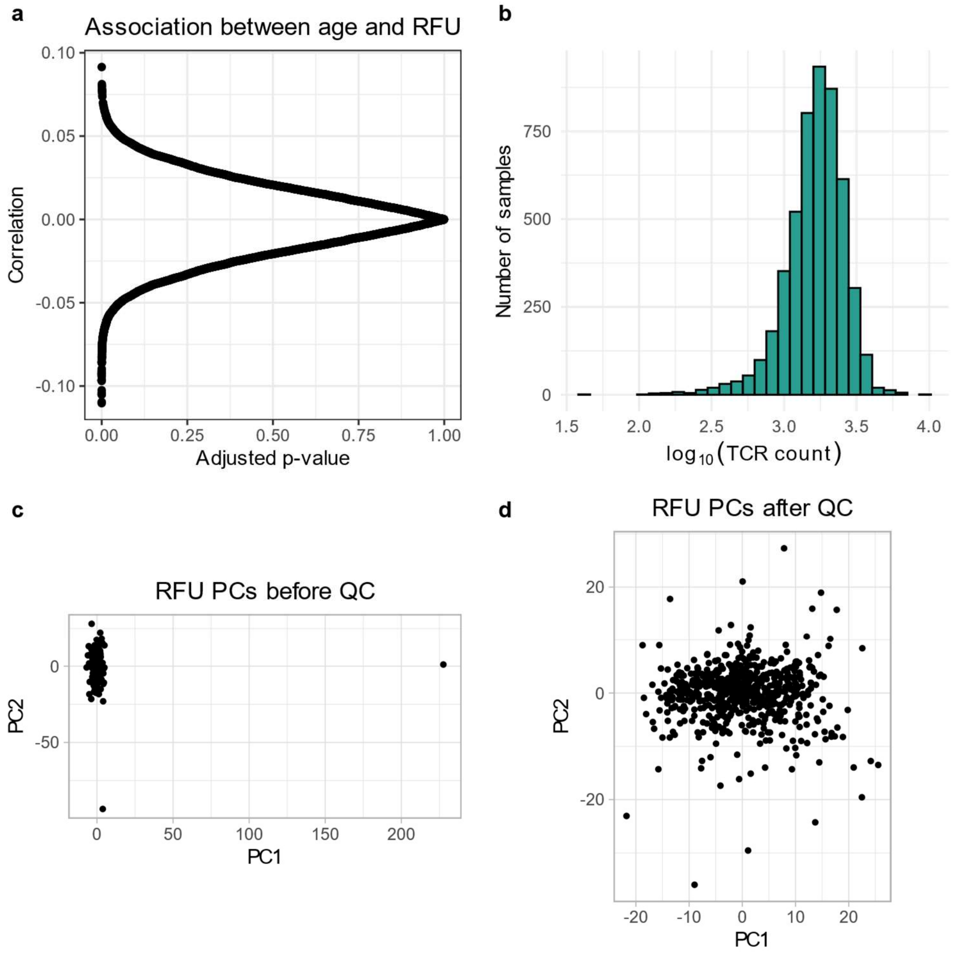

Supplementary Fig. 2 | rfuQTL training set and QC. **a**, the Spearman correlation and adjusted p-value of the associations between sample collection age and RFU abundances, restricting to samples containing over 1,500 unique CDR3 sequences. **b**, the number of unique CDR3 sequences per sample. **c**, **d**, the first two PCs of RFU abundance before (**c**) and after QC (**d**).

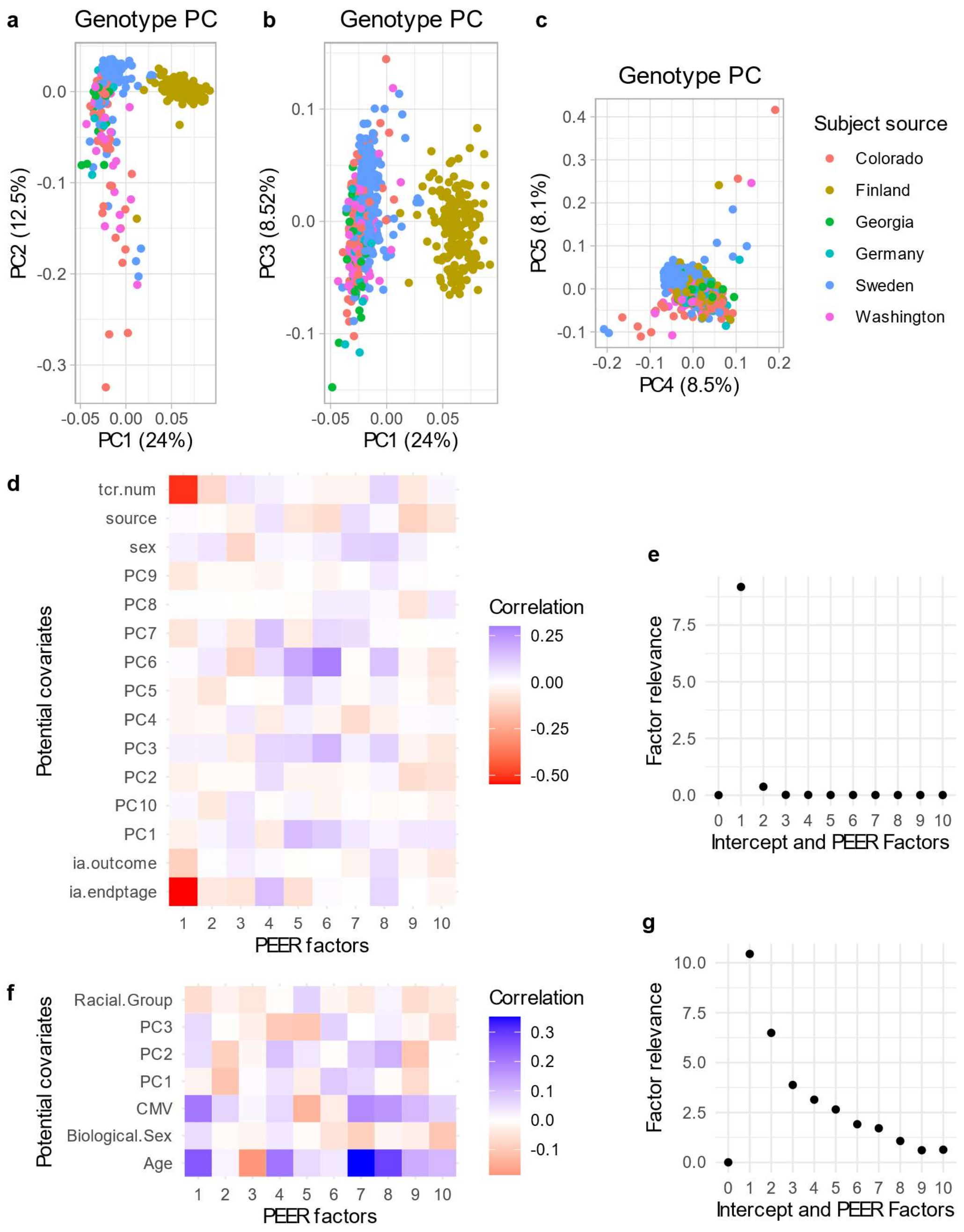

Supplementary Fig. 3 | Covariates in rfuQTL analysis. a, **b**, **c**, the first five genotype PCs, stratified by subject sources. The variance explained by each PC is indicated in parentheses. **d**, **f**, the Pearson correlation between the first ten PEER factors and potential covariates in the training set (**d**) and test set (**f**). **e**, **g**, the variance in RFU abundances explained by the intercept and PEER factors in the training set (**e**) and test set (**g**).

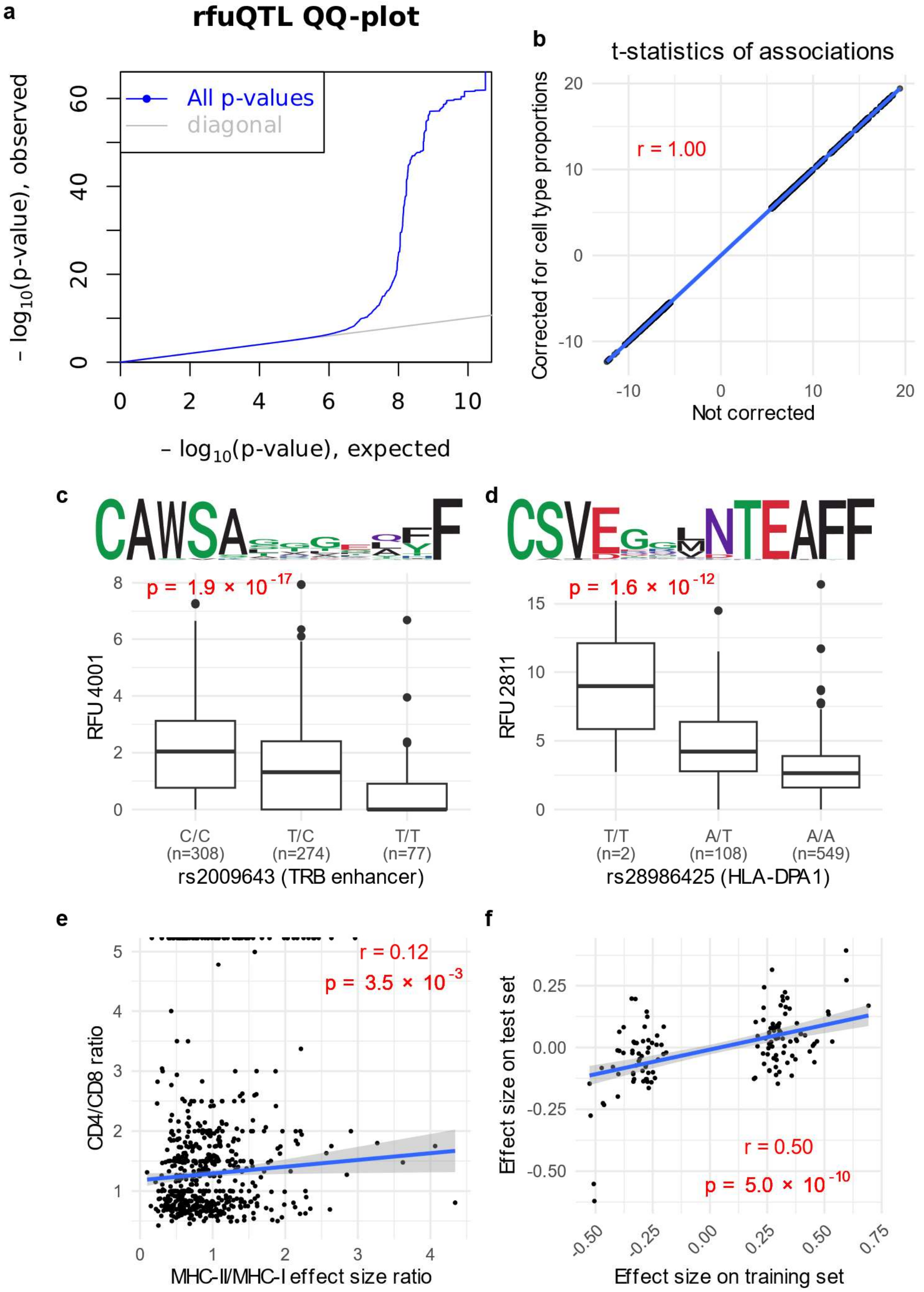

Supplementary Fig. 4 | Genetic variants and HLA haplotype associated with RFU abundances. a, quantile-quantile plot of p-values of associations between genetic variants and RFU abundances. The diagonal line represents expected p-values under the null hypothesis of no genetic associations. **b**, the t-statistics of associations between genetic variants and RFU abundances with cell type proportions corrected and not corrected. **c**, **d**, examples of variants-RFU associations. The top of each panel displays the RFU motif, while the bottom part illustrates the unnormalized RFU abundances corresponding to different variants. **e**, the cell type ratio, calculated as the mean CD4/CD8 ratio from two sorted TCR-seq datasets, and the effect size ratio derived from the strongest associations between the RFU and all HLA haplotypes categorized under MHC-II or MHC-I. Given that MHC-II antigens are recognized by CD4 T cells, and MHC-I antigens by CD8 T cells, a positive correlation between the CD4/CD8 ratio and the MHC-II/MHC-I effect ratio is expected. **f**, The effect size of the associations between HLA haplotypes and normalized RFU abundances for both training and test sets. The top five most significant associations for each HLA haplotype in the training set were displayed.

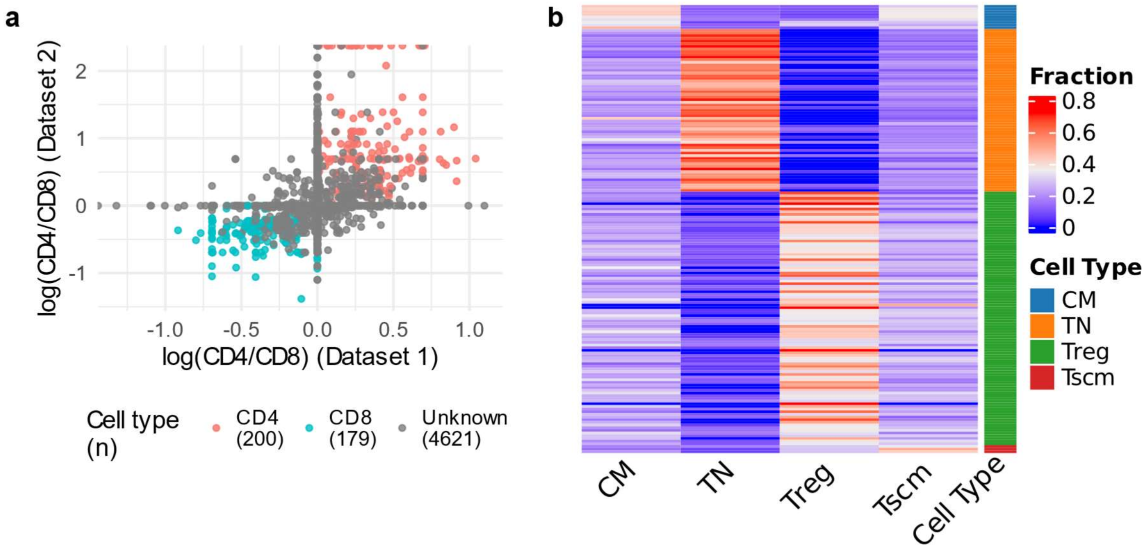

Supplementary Fig. 5 | RFU annotations. a, the CD4 to CD8 ratio for 5,000 RFUs derived from two sorted TCR-seq datasets. RFUs were labeled as CD4 or CD8 based on consistent enrichment across both datasets. **b**, the median fraction of CM, TN, Treg, Tscm within each RFU across all individuals. Each row represents an individual RFU, annotated with the cell type exhibiting the highest median abundance. Only RFUs with significant differences among T cell subsets are shown.

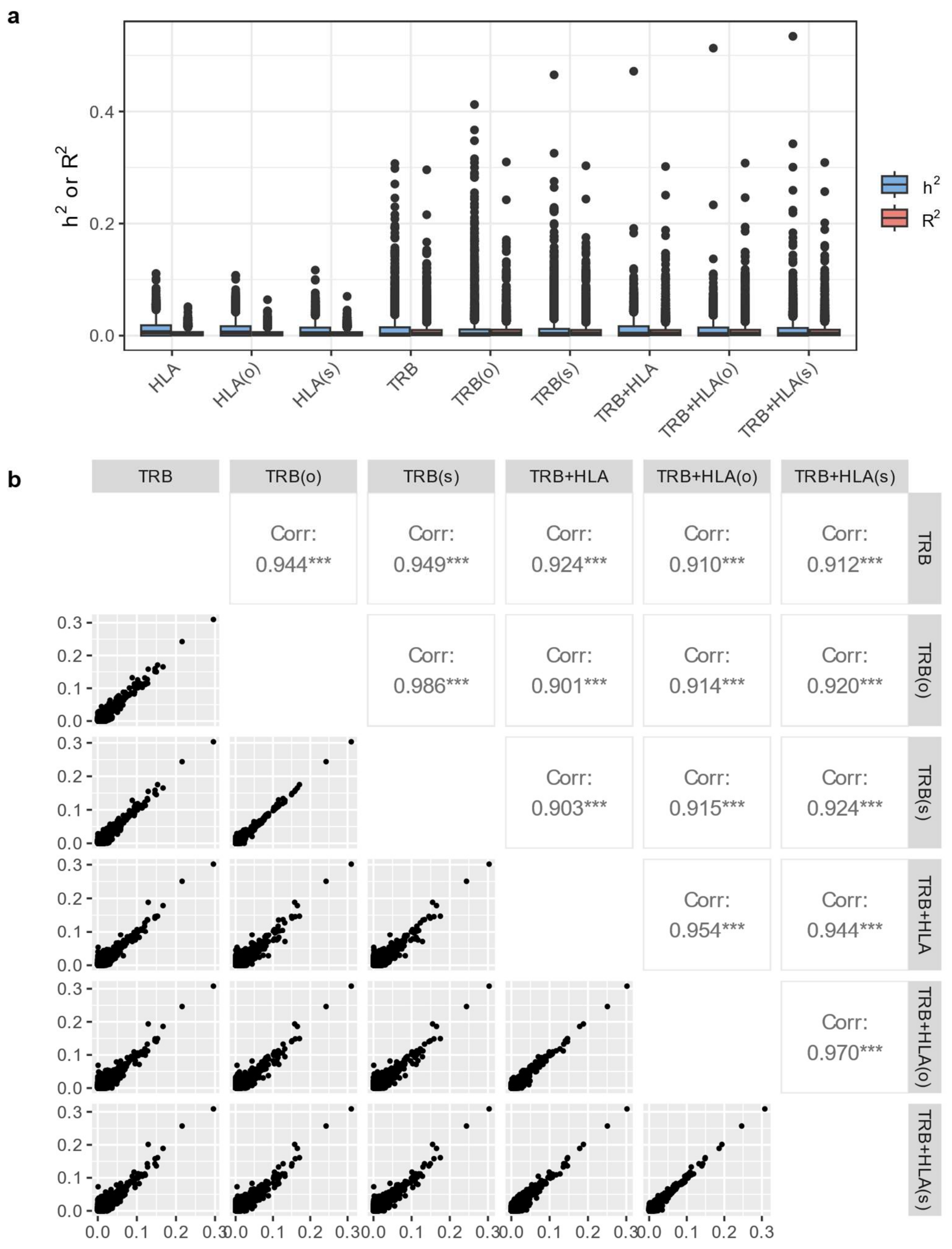

Supplementary Fig. 6 | Predictive models for RFU abundances. a, the heritability and cross-validated R^2 of lasso models, trained on various variant sets including TRB locus, HLA locus, and a combined TRB+HLA locus. For each locus, considered variants were within a 1Mb window, specific to the gene locus (denoted with “o”), and within variant windows defined by the first and last variant showing significant associations with at least one RFU ($P < 5 \times 10^{-8}$; denoted with “s”). **b**, the performance comparison of different variant sets, specifically focusing on TRB and TRB+HLA loci due to their superior performance as shown in a. *** is denoted if the correlation has a *P*-value less than 0.001.

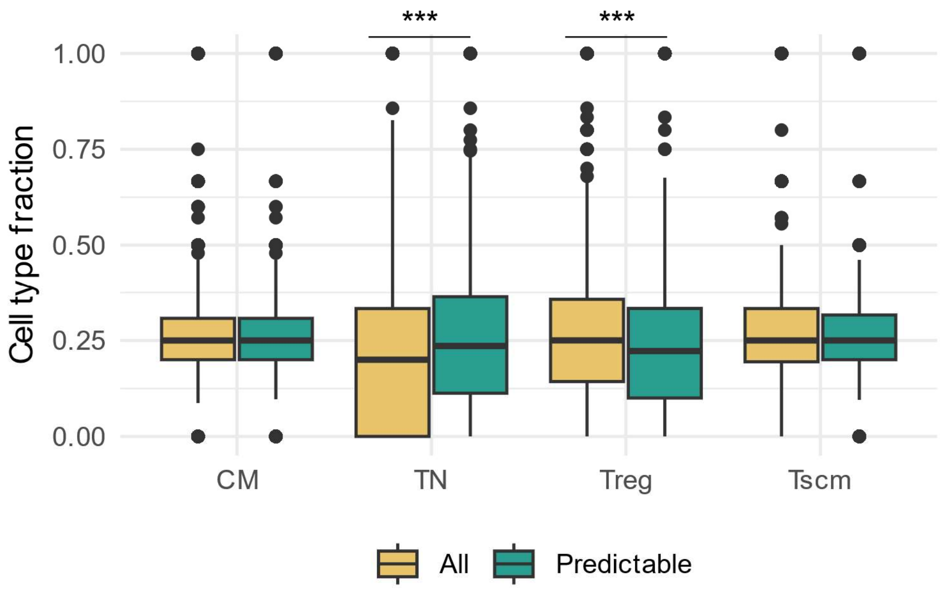

Supplementary Fig. 7 | The proportion of each cell type within each RFU, comparing the distribution across all RFUs and predictable RFUs. Significance is evaluated using Wilcoxon signed-rank test, with *** indicating $P < 0.0001$

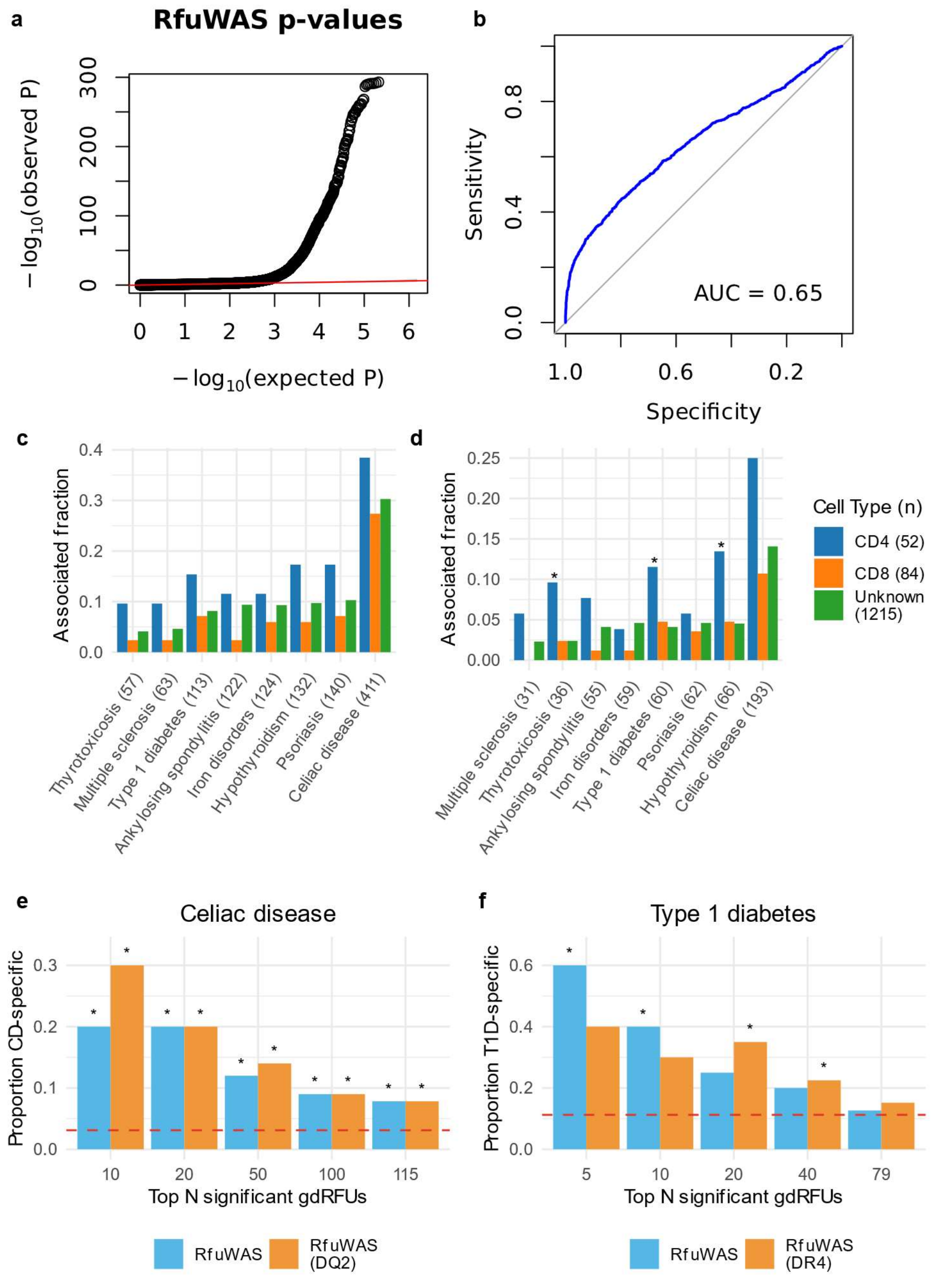

Supplementary Fig. 8 | gdRFUs associated with diseases. a, quantile-quantile plot of p-values of associations between gdRFU abundances and diseases. The diagonal line represents expected p-values under the null hypothesis of no associations. **b**, we repeated the benchmark in the RFU manuscript on another 896 TCR sequences specific to ten randomly selected epitopes. For each pair of TCR sequences, we calculated the Euclidean distance of their embedding as the predictor, with the response being if or not the two TCRs share the same antigen. We achieved an AUROC of 0.65, which is similar to the 0.64 in our previous benchmark. **c**, **d**, the fraction of disease-associated RFUs (**c**) and pathogenic RFUs (**d**) within CD4 and CD8 T cells. The numbers of associated RFUs are indicated in parentheses following each disease type, and the numbers of RFUs per cell type are indicated in parentheses following each cell type. **e**, **f**, the proportion of antigen-specific RFUs for the top N significant RFUs identified in RfuWAS for all individuals and individuals with HLA-DQ2 (**e**) or HLA-DR4 (**f**) haplotypes. The red dashed lines indicate the expected proportion of antigen-specific RFUs under random prediction. Significance is assessed using Fisher's exact test, with * denoting $P < 0.05$.

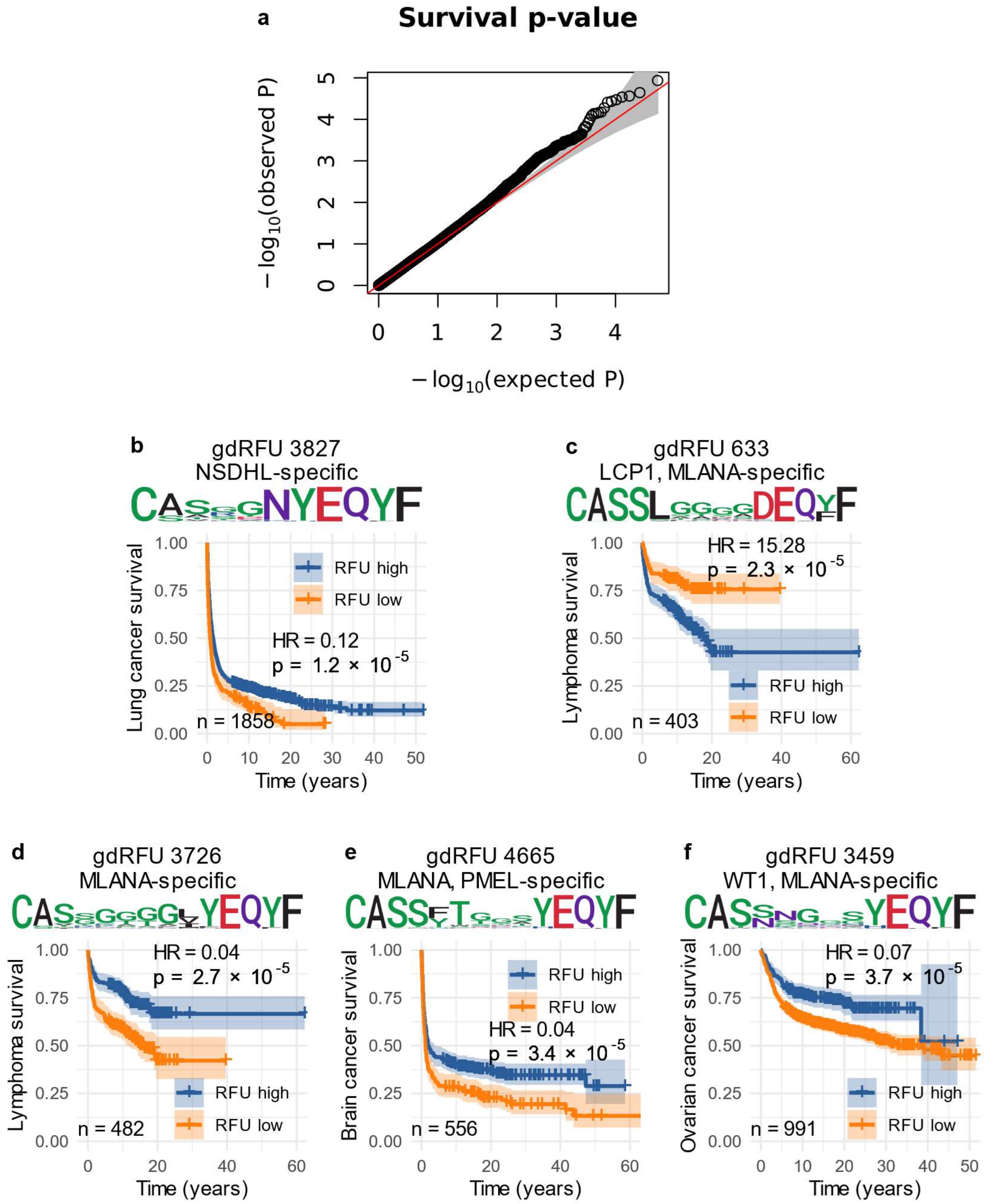

Supplementary Fig. 9 | gdRFUs associated with cancer survival. a, quantile-quantile plot of p-values of associations between gdRFU abundances and cancer survival. The diagonal line represents expected p-values under the null hypothesis of no associations, and the gray shade represents the 95% confidence interval. **b**, **c**, **d**, **e**, **f**, Kaplan–Meier survival curves illustrating the impact of gdRFUs on cancer outcomes. Each panel details the specific RFU number, associated antigens, and motif. The threshold for classifying RFU as "low" or "high" was determined using maximally selected rank statistics from the "survminer" R package. **b**, cancer of bronchus; lung (phecode 165.1). **c**, **d**, DLBCL (phecode 202.24, ICD 10 code C83.3). **e**, malignant and unknown neoplasms of brain and nervous system (phecode 191). **f**, malignant neoplasm of ovary and other uterine adnexa (phecode 184.1). *P*-values and hazard ratios (HR) were estimated using Cox proportional hazards models, with corrections for diagnose age, sex, and ancestry.

Supplementary Table 1: Characteristics of rfuQTL and rfuWAS datasets

		rfuQTL training set	rfuQTL test set	rfuWAS dataset
Reference		TEDDY	Emerson et al.	UK Biobank
TCR data	Sequencing method	RNA-seq (longitudinal)	TCR-seq	NA
	Material	Messenger RNA	DNA	
	n of unique CDR3s per individual, mean (s.d.)	11,304 (12,349)	242,461 (102,355)	
Genotype data	Sequencing method	WGS	Microarray with imputation	Microarray with imputation
	n of samples with genome-wide genotype	659	398	337,122
HLA haplotype data	Sequencing method	Imputed	Direct typing	Imputed
	n of samples with HLA haplotype	659	628	337,122
Phenotype data		Not relevant	Not relevant	1,086 phecodes
Ancestry	Identification method	Computed	Self reported	Self reported
	European, n	627	348	337,122
	Latin American, n	22	0	0
	Asian-Pacific Islander, n	2	19	0
	South Asian, n	2		0
	African American, n	1	8	0
	Native American or Alaska Native, n	0	8	0
	Unknown, n	5	245	0
Age, mean (s.d.)		1.7 (1.2)	39.5 (14.0)	56.9 (8.0)
Sex, n	Male	297	324	156,105
	Female	362	282	181,017
	Unknown	0	22	0

Supplementary Table 2: Significant associations between HLA haplotypes and normalized RFUs (rfuQTL training set)

SNP	RFU	beta	t.stat	p.value
HLA_DPB1*04:02	2811	0.69253	7.167129	2.07E-12
HLA_DQB1*02:01	815	-0.33356	-6.65596	5.94E-11
HLA_DQA1*05:01	815	-0.32988	-6.57972	9.63E-11
HLA_DRB1*03:01	815	-0.3201	-6.22523	8.57E-10
HLA_DRB1*08:01	1403	0.708504	6.164888	1.23E-09
HLA_DQB1*02:01	471	0.306704	5.940229	4.62E-09
HLA_DQA1*05:01	471	0.302953	5.865568	7.09E-09
HLA_DQA1*04:01	1403	0.594568	5.778948	1.16E-08
HLA_DQB1*04:02	1403	0.596177	5.774847	1.19E-08
HLA_B*08:01	471	0.317779	5.677258	2.06E-08
HLA_DRB1*03:01	471	0.298627	5.645551	2.45E-08
HLA_DRB1*03:01	4022	0.287664	5.430183	7.93E-08
HLA_DQB1*02:01	4022	0.279668	5.393606	9.65E-08
HLA_B*08:01	4022	0.302211	5.38788	9.94E-08
HLA_DQA1*05:01	3671	-0.27988	-5.34898	1.22E-07
HLA_DQA1*05:01	4022	0.275277	5.306894	1.53E-07
HLA_C*07:01	471	0.288563	5.23783	2.19E-07
HLA_DQB1*02:01	3671	-0.2734	-5.2183	2.42E-07
HLA_DRB1*03:01	3671	-0.27673	-5.16631	3.17E-07

Supplementary Table 3: Datasets in the study

Reference	Data modality	Tissue	Sample size	Utilization
rfuQTL training set	See Supplementary Table 1			rfuQTL of variants and HLA haplotype; lasso model training
rfuQTL test set	See Supplementary Table 1			rfuQTL of HLA haplotype; lasso model testing
rfuWAS dataset	See Supplementary Table 1			rfuWAS for diseases and cancer survival
Emerson et al.	TCR-seq	PBMC	42 individuals with sorted CD4 and CD8	CD4 and CD8 annotation dataset 1
Cader et al.	TCR-seq	PBMC	51 individuals with sorted CD4 and CD8	CD4 and CD8 annotation dataset 2
Gomez-Tourino et al.	TCR-seq	PBMC	14 healthy individuals and 14 T1D patients with sorted TN and CM; 8 healthy individuals and 8 T1D patients with sorted Treg and Tscm	TN, CM, Treg, and Tscm annotation dataset; T1D TCR-seq dataset
Lee et al.	TCR-seq	intraepithelial cell (IEC) and lamina propria cell (LPC)	7 healthy individuals and 22 CD patients with IEC; 6 healthy individuals and 18 CD patients with LPC	CD TCR-seq dataset
TCGA	RNA-seq	Multiple tissues	10,250 cancer patients with multiple types	DEG and GSEA between patients with and without protective RFUs

Supplementary Table 4: Cross-validated performance of RFU abundance prediction models

	Overlapping variants among three datasets		Overlapping variants between train and rfuWAS		Variants of training set	
Number of RFUs above R2 cutoff	Lasso	Elastic Net	Lasso	Elastic Net	Lasso	Elastic Net
All	4953	4953	4953	4953	4953	4953
0.01	1051	1037	1351	1330	1347	1338
0.04	97	97	160	155	169	172
0.09	23	21	37	37	37	36
