# Supplementary material for: Interpretable GWAS by linking clinical phenotypes to quantifiable immune repertoire components
Source: Commun Biol. 2024 Oct 20;7:1357. doi: 10.1038/s42003-024-07010-x (PMC11491462; doi:10.1038/s42003-024-07010-x)
Supplement: Supplementary file 3 — Description of Additional Supplementary Materials [file 42003_2024_7010_MOESM3_ESM.pdf]

Description of Additional Supplementary Files

File name: Supplementary Data 1-6

Description: The file includes Supplementary Data 1-6, and the specific descriptions for each tab are as follows. Supplementary Data 1: Significant associations between variants and normalized RFUs (rfuQTL training set). Supplementary Data 2: CD4 and CD8 annotation for RFUs. Supplementary Data 3: CM, TN, Treg, Tscm annotation for RFUs. Supplementary Data 4: Significant associations between predicted RFUs and phecodes (UKBB). Supplementary Data 5: RFU benchmark with ten epitopes. Supplementary Data 6: CD- and T1Dspecific TCR sequences in McPAS-TCR database
