# Supplementary material for: Interpretable GWAS by linking clinical phenotypes to quantifiable immune repertoire components
Source: Commun Biol. 2024 Oct 20;7:1357. doi: 10.1038/s42003-024-07010-x (PMC11491462; doi:10.1038/s42003-024-07010-x)
Supplement: Supplementary file 5 — Reporting Summary [file 42003_2024_7010_MOESM5_ESM.pdf]

nature portfolio

Corresponding author(s): Bo Li, Xiaowei Zhan, Dajiang Liu

Last updated by author(s): Mar 18, 2024

Reporting Summary

Nature Portfolio wishes to improve the reproducibility of the work that we publish. This form provides structure for consistency and transparency in reporting. For further information on Nature Portfolio policies, see our Editorial Policies and the Editorial Policy Checklist.

Statistics

For all statistical analyses, confirm that the following items are present in the figure legend, table legend, main text, or Methods section.

n/a	Confirmed	
☐	☒	The exact sample size (*n*) for each experimental group/condition, given as a discrete number and unit of measurement
☒	☐	A statement on whether measurements were taken from distinct samples or whether the same sample was measured repeatedly
☐	☒	The statistical test(s) used AND whether they are one- or two-sided<br>*Only common tests should be described solely by name; describe more complex techniques in the Methods section.*
☐	☒	A description of all covariates tested
☐	☒	A description of any assumptions or corrections, such as tests of normality and adjustment for multiple comparisons
☐	☒	A full description of the statistical parameters including central tendency (e.g. means) or other basic estimates (e.g. regression coefficient) AND variation (e.g. standard deviation) or associated estimates of uncertainty (e.g. confidence intervals)
☐	☒	For null hypothesis testing, the test statistic (e.g. *F*, *t*, *r*) with confidence intervals, effect sizes, degrees of freedom and *P* value noted<br>*Give P values as exact values whenever suitable.*
☒	☐	For Bayesian analysis, information on the choice of priors and Markov chain Monte Carlo settings
☒	☐	For hierarchical and complex designs, identification of the appropriate level for tests and full reporting of outcomes
☐	☒	Estimates of effect sizes (e.g. Cohen's *d*, Pearson's *r*), indicating how they were calculated

Our web collection on statistics for biologists contains articles on many of the points above.

Software and code

Policy information about availability of computer code

Data collection

No software was used for data collection.

Data analysis

TRUST4: https://github.com/liulab-dfci/TRUST4
GRAF-pop 1.0: https://www.ncbi.nlm.nih.gov/projects/gap/cgi-bin/GetZip.cgi?zip_name=GrafPop1.0.tar.gz
plink 2.0: https://www.cog-genomics.org/plink/2.0/
Michigan Imputation Server 1.2.4: https://imputationserver.sph.umich.edu/index.html#!
PEER 1.3: https://github.com/PMBio/peer.git
GCTA 1.94.1: https://yanglab.westlake.edu.cn/software/gcta/bin/gcta-1.94.1-linux-kernel-3-x86_64.zip
CreateUKBphenome: https://github.com/umich-cphds/createUKBphenome
R 4.2.3: https://www.r-project.org/
MatrixEQTL 2.3: https://cran.r-project.org/web/packages/MatrixEQTL/index.html
PrediXcan 0.6.11: https://github.com/hakyimlab/MetaXcan
GSEA 4.3.2: https://www.gsea-msigdb.org/gsea/index.jsp
EnrichmentMap 3.3.5: https://enrichmentmap.readthedocs.io/en/latest/
AutoAnnotate 1.4: https://autoannotate.readthedocs.io/en/latest/
RfuWAS: https://github.com/YuhaoTan2/RfuWAS

For manuscripts utilizing custom algorithms or software that are central to the research but not yet described in published literature, software must be made available to editors and reviewers. We strongly encourage code deposition in a community repository (e.g. GitHub). See the Nature Portfolio guidelines for submitting code & software for further information.

Data

Policy information about availability of data

All manuscripts must include a data availability statement. This statement should provide the following information, where applicable:

- Accession codes, unique identifiers, or web links for publicly available datasets
- A description of any restrictions on data availability
- For clinical datasets or third party data, please ensure that the statement adheres to our policy

Raw data analyzed in this study are available at the following locations: dbGaP: phs001442, phs001918; ImmuneAccess database: https://doi.org/10.21417/B7001Z, https://doi.org/10.21417/B7H01M, https://doi.org/10.21417/B7C88S, https://doi.org/10.21417/LWL2022JCP. Access to UK Biobank individual-level data can be requested from https://www.ukbiobank.ac.uk/enable-your-research/apply-for-access. The weights of the lasso models are deposited at GitHub: https://github.com/YuhaoTan2/RfuWAS/blob/main/models/lasso_weights_tsv.zip.

Research involving human participants, their data, or biological material

Policy information about studies with human participants or human data. See also policy information about sex, gender (identity/presentation), and sexual orientation and race, ethnicity and racism.

Reporting on sex and gender	Findings apply to both sexes. We analyze males and females together in the study. Sex was determined based on self-reporting and confirmed based on their genotype. Sex in cohorts: 297 males and 362 females in rfuQTL training set; 324 males, 282 females, and 22 unknowns in rfuQTL test set; 156,105 males and 181,017 females in rfuWAS dataset.
Reporting on race, ethnicity, or other socially relevant groupings	We used genotype PCs in the study to control for confounding variables. We only used self-reported race/ethnicity or computed ancestry when reporting the summary characteristics. See Supplementary Table 1 for details.
Population characteristics	The mean and standard deviation of ages in cohorts: rfuQTL training set: mean 1.7, sd 1.2; rfuQTL test set: mean 39.5, sd 14.0; rfuWAS dataset: mean 56.9, sd 8.0. Sex and genotype PCs are described above.
Recruitment	Participants were recruited in previous studies.
Ethics oversight	Data collection were performed in previous studies.

Note that full information on the approval of the study protocol must also be provided in the manuscript.

Field-specific reporting

Please select the one below that is the best fit for your research. If you are not sure, read the appropriate sections before making your selection.

☒ Life sciences ☐ Behavioural & social sciences ☐ Ecological, evolutionary & environmental sciences

For a reference copy of the document with all sections, see nature.com/documents/nr-reporting-summary-flat.pdf

Life sciences study design

All studies must disclose on these points even when the disclosure is negative.

Sample size	The sample size was determined based on the number of samples that are publicly available. We used 659 samples in rfuQTL training set, 398 samples in rfuQTL test set, and 337,122 samples in rfuWAS dataset.
Data exclusions	For rfuQTL training set, we excluded individuals who deviated by more than ±3 standard deviation in heterozygosity rate from the mean, those exhibiting high relatedness (defined as a Kinship-based INference for Genome-wide association studies (KING) coefficient greater than 0.0884), and individuals with fewer than 2,500 unique CDR3 beta chains. For RfuWAS dataset, the study cohort was restricted to unrelated individuals of white British ancestry, with unrelatedness defined as used in principal component analysis (PCA) calculation. Additional inclusion criteria included the absence of putative sex chromosome aneuploidy and the availability of genotype data. These quality controls are common practice in GWAS analysis to correct for covariates.
Replication	We replicated the genetic associations of RFU and the predictive models in an independent test set.
Randomization	Randomization is not applicable to genetic association studies in case control and population-based biobanks.
Blinding	Blinding is not applicable to genetic association studies in case control and population-based biobanks.

Reporting for specific materials, systems and methods

We require information from authors about some types of materials, experimental systems and methods used in many studies. Here, indicate whether each material, system or method listed is relevant to your study. If you are not sure if a list item applies to your research, read the appropriate section before selecting a response.

Materials & experimental systems

n/a	Involved in the study
☒	☐ Antibodies
☒	☐ Eukaryotic cell lines
☒	☐ Palaeontology and archaeology
☒	☐ Animals and other organisms
☒	☐ Clinical data
☒	☐ Dual use research of concern
☒	☐ Plants

Methods

n/a	Involved in the study
☒	☐ ChIP-seq
☒	☐ Flow cytometry
☒	☐ MRI-based neuroimaging

Plants

Seed stocks	*Report on the source of all seed stocks or other plant material used. If applicable, state the seed stock centre and catalogue number. If plant specimens were collected from the field, describe the collection location, date and sampling procedures.*
Novel plant genotypes	*Describe the methods by which all novel plant genotypes were produced. This includes those generated by transgenic approaches, gene editing, chemical/radiation-based mutagenesis and hybridization. For transgenic lines, describe the transformation method, the number of independent lines analyzed and the generation upon which experiments were performed. For gene-edited lines, describe the editor used, the endogenous sequence targeted for editing, the targeting guide RNA sequence (if applicable) and how the editor was applied.*
Authentication	*Describe any authentication procedures for each seed stock used or novel genotype generated. Describe any experiments used to assess the effect of a mutation and, where applicable, how potential secondary effects (e.g. second site T-DNA insertions, mosiacism, off-target gene editing) were examined.*
